# Whole-Genome Sequence of a Strain of Brucella melitensis Isolated from a Patient with Swelling of the Right Testicle in Inner Mongolia, China

**DOI:** 10.1128/MRA.00110-21

**Published:** 2021-05-20

**Authors:** Shu-yi Ma, Miao Wang, Jone Ngobeh, Zhi-guo Liu, Zhen-jun Li

**Affiliations:** a School of Medical Technology, Baotou Medical College, Baotou, China; b Ulanqab Center for Disease Control and Prevention, Jining, China; c Sierra Leone-China Biosafety Laboratory, Jui, Freetown, Sierra Leone; d State Key Laboratory for Infectious Disease Control and Prevention, National Institute for Communicable Disease Control and Prevention, Chinese Center for Disease Control and Prevention, Beijing, China; Loyola University Chicago

## Abstract

This report describes the isolation, sequencing, and annotation of Ws20160810, which was isolated from a blood sample from a brucellosis patient suffering from swelling of the right testicle in the Inner Mongolia Autonomous Region, China. The genome size was 3,244,234 bp with a 57.23% GC content, 3,294 coding DNA sequences (CDSs), 55 tRNAs, 5 rRNAs (5S [*n* = 2], 16S [*n* = 1], and 23S [*n* = 2]), and 3 small RNAs (sRNAs).

## ANNOUNCEMENT

Brucellosis can be transmitted from animal reservoirs to humans through direct or indirect contact with infected animals or contaminated products (1). Brucella melitensis is the predominant species among circulating strains of Brucella in China and the primary species that causes brucellosis in the country (2–4). Brucella species are common animal pathogens, causing economic losses through spontaneous abortion and decreased milk production in livestock (5). Fever, sweats, chills, and joint pain are the main manifestations in humans. In men, testicular pain and swelling are among the most common symptoms (6). Here, whole-genome sequencing was performed on a strain obtained from a patient with swelling of the right testicle. The Ethics Committee of the National Institute for Communicable Disease Control and Prevention, Chinese Center for Disease Control and Prevention, approved the research protocol. The ethics approval number is ICDC-2018005.

Culture and isolation of the pathogen were performed as described previously (7, 8). A total of 5 ml of blood was collected, injected into diphase culture bottles (Bio-Merieux, Beijing, China), and incubated at 37°C under 5% CO_2_ conditions for 2 weeks. The isolate, which we named Ws20160810, was identified as Brucella melitensis based on biotyping methods (9). Prior to the isolation of genomic DNA, a single colony was subcultured on Brucella agar plate medium at 37°C for 48 h. Subsequently, genomic DNA was extracted using a QIAamp DNA minikit (Qiagen, Germany) following the manufacturer’s instructions. Sequencing libraries were generated using the NEBNext Ultra DNA library prep kit for Illumina (NEB, USA) following the manufacturer’s recommendations. The library size was 350 bp, and a paired-end (PE) sequencing strategy (2 × 150 bp) was used on the Illumina NovaSeq PE150 platform to determine the whole-genome sequence of Ws20160810. Sequencing yielded a total of 1,116 Mb raw data reads, which were trimmed using Trimmomatic v. 0.38 (10) to yield a total of 1,000 Mb clean reads following the removal of Illumina adaptor sequences and low-quality reads. The reads had an average length of 150 bp, with more than 94% of the bases having a quality score above Q30. The clean reads were assembled using SOAPdenovo v. 2.04 (11, 12) into 27 contigs (24 scaffolds) that were each at least 200 nucleotides (nt) long, for a total of 3,295,722 bp with a GC content of 57.23%, an *N*_50_ value of 299,257, and an *N*_90_ value of 116,358 nt. The genome coverage was 100% (coverage = size covered by sequencing reads/total genome size × 100%). The sequencing depth was calculated to be 303× using the following formula: sequencing depth = total number of bases generated (1,000 Mb)/size of genome sequenced (3.3 Mb). The annotation was performed using RNAmmer v. 1.2 (13), tRNAscan-SE v. 1.3.1 (14), TRF v. 4.07b (15), DFAST v. 1.2.4 (16), and GeneMarkS v. 4.17 (17) with default settings. The genome consisted of 3,294 coding sequences (CDSs), 55 tRNAs, 5 rRNAs (2 5S, 1 16S, and 2 23S rRNAs), and 3 small RNAs (sRNAs). Based on the NCBI SRA Taxonomy Analysis Tool (STAT), the genome sequence of strain Ws20160810 was 97.4% similar to the sequence of B. meliteneis strain 16M. Further comparative genome analysis of the strain will provide valuable insight for better understanding of the pathogenic characteristics of the strain.

### Data availability.

The whole-genome sequence of B. melitensis strain Ws20160810 was deposited in GenBank under the accession number JAEUGC000000000. The raw sequence data are available under the SRA accession number SRR13558868. The BioProject accession number is PRJNA694541, and the BioSample accession number is SAMN17526370.
